# Socio-emotional correlates of a schooldog-teacher-team in the classroom

**DOI:** 10.3389/fpsyg.2013.00886

**Published:** 2013-11-27

**Authors:** Andrea Beetz

**Affiliations:** Department of Education, University of Erlangen-NurembergErlangen, Germany

**Keywords:** animal-assisted education, classroom, dog, emotion regulation, school, children

## Abstract

A growing number of teachers in Europe regularly take their dogs with them into the classroom. Limited research points at positive socio-emotional effects of this practice. In this study the effects of a schooldog-teacher-team on socioemotional experiences in school, depression and emotion regulation strategies were investigated in a classroom of third-graders (male *n* = 12, female *n* = 13), which had a schooldog present for 1 day per week in comparison with a control class (male *n* = 11, female *n* = 10). In contrast to the control class, the dog-class students reported a stronger improvement with regard to *positive attitude toward school* (repeated measures ANOVA; *F* = 10.769, *df* = 1, *p* = 0.002) and *positive emotions related to learning* (*F* = 4.479, *df* = 1, *p* = 0.042) over the course of the year. Since a prerequisite of all kinds of effective learning is a positive attitude and mood toward school and learning, the presence of a schooldog-teacher team thus has the potential to support learning.

## Introduction

During the last decade it has become increasingly popular among German, Austrian, and Swiss teachers to take their dogs into the classroom (Agsten et al., 2011; Beetz, 2012). In contrast to dogs visiting the classroom with a handler only on one or two occasions to teach the children about safe behavior toward dogs (“school-visiting dogs”), a dog owned by the teacher and brought to the school regularly (1–5 days per week) is commonly referred to as “schooldog”. Agsten (2009) observed an exponential increase of “schooldog-teacher-teams” that registered in a special network for teachers with schooldogs (www.schulhundweb.de) over the previous 10 years. It can be assumed that this trend prevailed during the last 5 years, also due to frequent media coverage in the German-speaking domain (Beetz, 2012). Based on this trend and 200 registrations in 2009 (Agsten, 2009) it seems likely that several hundred (>500) teachers in the three countries work with their dogs in schools on a regular basis. This estimate also pays respect to the fact that many teachers working with schooldogs do not register in this network, since registration is voluntary. No data on the number of schooldogs are provided by the authorities, since the general decision of admitting a schooldog is made by the headmaster of the school. The permission of allowing a schooldog into a specific classroom of students also depends on parental consent and the absence of contraindications such as allergies to dogs.

### The practice of animal-assisted education with schooldogs

Keeping companion animals in the classroom is not an uncommon practice in Western Societies. Rud and Beck (2000) conducted a survey asking over 400 teachers in the USA about the goals they pursue with this practice. More than just wanting to improve the atmosphere, teachers experienced the animals to be subjects for academic research as well as prompts for creative activities in the classroom. Furthermore, animals were used by students to calm themselves, to improve psychological wellbeing, and to contribute to enjoyment in the school setting. A survey of 37 teachers in elementary schools in the USA by Zasloff et al. (1999) also points to the role of animals in the classroom for teaching humane attitudes and values, and for motivating students with learning problems and other difficulties.

Students with emotional problems reported in a survey by Robin et al. (1983) that companion animals provided them with having someone to love, while students from regular schools rather focused on learning to take responsibility for a living being.

In contrast to the studies reported above, which included different kinds of animals kept in the classroom or by students, the following research addressed the presence of dogs in schools.

A survey of 77 teachers working with schooldogs (Beetz and Marhofer, 2012a) provided some insight into this practice of dog-assisted education: Most frequently (>40%) schooldogs were present in primary schools (grades 1–4), followed by schools for special education (>30%). One third of dogs were present for only 1 day per week, about 50% for 2–3 days per week, usually in the morning. Most of the time, the dog was just present in the classroom, was allowed to move around freely or also to rest wherever it chose. Usually, the dog was actively involved in the instruction several times per day for short intervals, for example by selecting a mathematical task via throwing a big soft dice, by distributing test results in a basket, or by doing little tricks with some children as reward for good cooperation of the class. In addition, some dogs supported certain students during difficult tasks, being close during tests or while practicing reading in a corner. More rarely, and usually in a special education setting, the direct work with the dog such as passing an agility course, obedience training, tricks or retrieval tasks was used to enhance coordination, self-control and to promote experiences of self-efficacy (Beetz, 2012).

Also in programs conducted by teachers for children with special needs, such as low reading ability, empathy, and social competence or difficulties with concentration, schooldogs are involved. Via its presence and free interaction with the children the dog potentially promotes the establishment of a trusting student-teacher-relationship and probably further supports socio-emotional and cognitive learning by reducing stress levels of the children (Beetz et al., 2011, 2012a,b; Julius et al., 2013).

Breeds of dogs involved vary widely. Most popular are Retrievers (ca. 30%), followed by mongrels (ca. 20% Beetz and Marhofer, 2012a). Also the training of the dogs or the schooldog-teacher-teams varies (Beetz and Marhofer, 2012a). It ranges from no special training to 2-year studies in animal-assisted interventions plus a certification of the dog as a therapy-dog. However, at the moment, no governmentally acknowledged certification procedure for these dogs or teachers exists. First official training-courses for schooldog-teacher-teams started in fall 2012 in Austria organized by two pedagogical universities.

### Goals and correlates of working with a schooldog

Most teachers working with schooldogs intend to influence the social behavior, socio-emotional competence, and empathy of their students (Beetz and Marhofer, 2012b), but also enjoyment of interacting with dogs and doing so safely. Further goals are improvement of class climate, motivation and discipline, since dogs provide a calm atmosphere and are also used as rewards and motivators. However, little research on the actual effects of dogs in the classroom exists to back these intended effects.

Hergovich et al. (2002) investigated the effects of the daily presence of one of three schooldogs in a classroom of first-graders for the duration of 3 months in comparison to a control class. The authors reported a significant improvement in empathy toward animals, increased field independence (a factor related to the cognitive aspect of empathy, based on perception of figures against a background) and a better integration of students in the class. No improvement in social intelligence and sociability was found. Of four children in the dog-class, who were identified as aggressive at the beginning of the project, two had improved remarkably, while there were no changes regarding the three aggressive children in the control-class. Overall, Hergovich et al. (2002) argued that the dogs improved the social climate and also helped to reduce aggression in some of the students. However, since other influences which could contribute to the differences between classes, such as teacher personality and teaching style were not controlled, the findings need to be interpreted with care.

In general, it is difficult with such a research design to clearly interpret changes in the children's experiences, test scores or behaviors as effects of the presence of a dog. Due to the comparison of different classrooms with different teachers any changes represent rather correlates of the presence of the schooldog-teacher team, than just the dog by itself. The dog may also affect the teacher, his or her behavior and mood, and rather contribute to the overall class climate.

In the “dog-class” investigated by Hergovich et al. (2002), Kotrschal and Ortbauer (2003) compared the behavior of the children before the introduction of the schooldogs with their behavior one month later. In the presence of the dogs, the children spent less time alone and more time in contact with others. Verbal provocations decreased and attention toward the teacher increased in the presence of the dogs. In addition, all forms of aggressive behavior occurred less frequently when the dogs were present. Children spent about 10% of the time in contact with the dog and in particular extreme behavioral tendencies, such as a high level of aggression of male children seemed to be attenuated. Qualitatively monitoring a classroom with six children diagnosed with severe emotional disorders, Anderson and Olson (2006) emphasized that the presence of the dog over a period of 8 weeks promoted emotional stability and the attitude toward school, helped to prevent and to de-escalate emotional crises, and supported the children's learning about responsibility, empathy and respect. While these subjective observations from a small group of children need to be interpreted with care, they correspond with the findings of quantitative studies reported above.

In the framework of a more rigorous study design and a relatively large sample, Tissen et al. (2007) investigated the additional effects of integrating dogs in a training of social competence. The training was conducted once per week for 90 min. In three schools three classes each, a total of 230 children, received one of the following interventions: social training with dogs; social training without dogs; dog presence without social training. Irrespective of the intervention children improved in social behavior and empathy. Via a standardized questionnaire children in the dog-assisted training reported a decline of open and relational aggression which was not the case in the other two interventions.

### Short-term effects of the presence of dogs in educational settings

Due to the scarcity of studies investigating the effects of the regular presence schooldogs directly, the following research on short-term effects applying an experimental design conducted by Gee and colleagues in a pre-school setting seems of interest. Preschoolers with and without developmental delays performed motor tasks faster, but with the same accuracy in the presence of a therapy dog in comparison to its absence (Gee et al., 2007). Also, children with and without speech problems needed fewer prompts in imitation tasks in the presence of a dog in comparison to the presence of an adult or a toy dog (Gee et al., 2009). Similarly, preschoolers needed less help with memory tasks and showed better concentration in the presence of a dog (Gee et al., 2010b). Furthermore, preschoolers made less mistakes in a sorting task in the presence of a dog (Gee et al., 2010a). Overall, this series of experiments suggests that the presence of a calm and friendly dog can promote concentration, but maybe also motivation while contributing to a more relaxed state and a reduction of stress in educational activities.

The presence of a dog, and in particular physical contact via stroking, can buffer and reduce the physiological stress reactions in response to a stressful, school-related task more effectively than the presence of a human, in particular in male children with insecure attachment representations which they developed via suboptimal experiences with their primary caregivers (Beetz et al., 2011, 2012a,b).

### Theoretical background of positive effects of schooldogs

Overall, dogs seem to have the potential to positively affect social behavior, in particular aggression, concentration, and physiological stress reactions of children in school-settings or with respect to school-related tasks. But what are the underlying mechanisms of these effects and are there other factors which could be positively affected by the presence of a schooldog?

An optimal level of activation or an adequate regulation of stress plays an important role with regard to socio-emotional, but also cognitive performance and learning (Beetz et al., 2012b; Julius et al., 2013). In particular the so-called “executive functions” (Miyake et al., 2000: concentration, impulse control, memory, cognitive flexibility, self-motivation, self-reflection, problem solving, and strategic planning), are regulated via the prefrontal cortex and react sensitively to elevated levels of cortisol (Diamond and Lee, 2011). The reduction of stress in children would explain short-term improvements in executive functions and thus performance in test situations. Low stress levels and good executive functions will contribute to a better social atmosphere in the classroom, since socially competent behavior is also based on good impulse control. This is probably the key executive function, especially with regard to socially incompetent aggressive impulses as well as strong withdrawal, which represent behavioral strategies for dealing with negative emotions such as fear, sadness or anger (Grob and Smolenski, 2009). The use of adaptive in contrast to maladaptive emotion regulation strategies has been linked to a better quality of life, including well-being, but also mental and physical health (Weber, 1997). Adaptive strategies are for example, seeking social support, problem-oriented actions, acceptance, re-appraisal or cognitive problem-solving. Among the maladaptive strategies are giving-up, aggressive behavior, withdrawal, self-devaluation and perseveration (Grob and Smolenski, 2009).

Today, there is a large body of research documenting the reduction of psychophysiological stress parameters via the presence of and contact with friendly animals, particularly dogs (for an overview see Beetz et al., 2012a). It was hypothesized (Beetz, 2012; Julius et al., 2013) that there may be different mechanisms involved, among them the promotion of calmness based on human biophilia and the activation of the oxytocin system. Human biophilia (Kellert and Wilson, 1993; Wilson, 1984) describes the interest in and attention toward animals and nature, based on human evolutionary history. Probably, animals were always cues for safety or danger (e.g., from predators). Therefore, calm and relaxed animals could promote human calmness, relaxation, and reduction of stress in humans even without direct physical contact (for details see Julius et al., 2013). Direct contact, for example via stroking the dog, is related to a reduction of stress parameters, which is probably mediated via the activation of the oxytocin system (Odendaal, 2000; Odendaal and Meintjes, 2003; Nagasawa et al., 2009; Handlin et al., 2011), a hormonal system linked to the promotion of calmness, social bonds, and the reduction of stress (Uvnäs-Moberg, 2003). Experimental research in humans and animals has documented that an increase of the level of oxytocin is linked to reduced physiological stress reactions, more calmness, reduced fear, depression, and aggression, and increased social orientation, interaction, attention, and trust (for an overview see Beetz et al., 2012b; Julius et al., 2013).

In parallel, research on human-animal interactions has shown similar effects of interactions with companion animals, in particular dogs (for an overview see Beetz et al., 2012b). This also applies to children. The research reported above documented potential positive effects of dogs on children with regard to aggression, attention, social interaction, and integration, motivation, and emotional self-regulation. Further studies found that the interaction with dogs was able to reduce depressive mood and to enhance positive affect (Kaminski et al., 2002) and intra-emotional balance (Prothmann et al., 2006).

### Research question

The main goal of this study was to investigate if the presence of a schooldog, or rather a schooldog-teacher-team, in a classroom of third-graders in elementary school could positively affect depressive mood, emotion regulation and social and emotional school experiences. We proposed a positive effect of the schooldog-teacher-team's regular presence on these factors based on previous research findings presented above. A potential improvement in mood, social behavior and stress, and via the link between stress and executive functions on emotion regulation and learning, should also be reflected in the students' general social and emotional school experiences.

It should be noted here that we expected effects based not only on the presence of the dog, but rather of how the dog and his teacher together as a team affected social interactions in the classroom.

## Material and methods

### Method

A class of third-graders which had a schooldog present for 1 day per week, and a control class without a dog from the same German elementary school were investigated. Before the dog was introduced in the third week after the beginning of the school-year (t1) and 2 weeks before the end of the school-year (t2) data were collected via standardized questionnaires. The classes included in the study were selected in accordance with the following criteria:

They had not had a schooldog before. It was possible to bring a dog into the classroom due to the absence of allergies in the dog-class. A suitable teacher with an experienced schooldog, who had been in classrooms for the previous 2 years, was not older than 9 years, healthy and had permission to visit classrooms by the school board and headmaster was willing to participate in the study. A teacher with a class in the same grade level at the same school, to control for socioeconomic influences, was willing to participate as well, together with the students.

The presence of the dog had been approved by the headmaster of the school and the authorities, who also reviewed the research proposal for ethical concerns regarding the children and the dog. No additional approval from the Ethics Committee (IRB) of the university was required, which is still not an unusual practice in Germany with regard to research in schools based on questionnaires, interviews or observations. Responsibility for the content of questionnaires, ethical conduct and obtaining informed consent lied with the principal investigator as did working with a schooldog-teacher team which adhered to animal welfare standards and regular checks by a veterinarian.

Informed consent of the authorities and all parents of the children involved in the study had been obtained and screening for allergies in the intervention class had been conducted at the end of the previous school year. The dog was a 7-year-old female Norwegian Lundehund that was regularly checked by a veterinarian and had a calm and friendly demeanor. It was completely secure with children of this age, even when approached by several children at a time. However, the teacher had established rules together with the students on how to approach the dog and how to behave when the dog was present. The dog was allowed to move around freely in the classroom and to rest wherever she liked; she had a quiet place behind the teacher's desk and free access to water at all times. The desks of the students were placed in a U-shape, and frequently the dog chose to rest in the free space in the middle. In addition, children were allowed to do little tricks with her and interact freely with her during class-time while observing the rules of conduct. The teacher was an experienced dog-owner with additional training in detecting stress-signals in dogs to secure the wellbeing of the animal in the classroom situation.

### Sample

The “dog-class” (intervention class) consisted of 12 male and 13 female children between 8 and 9 years of age (*M* = 8.5 years, *SD* = 0.51). Eleven male and ten female children represented the control class (*M* = 8.4, *SD* = 0.51). The majority of the children (>90%) came from a German-speaking, White Caucasian background. Three students of the control class (2 male, 1 female) and one male student of the intervention class missed the data collection at t2, and data could also not be obtained from them until the end of the year. These data were omitted in the analysis (original class sizes were *n* = 26 for the dog-class and *n* = 24 for the control class). The two main teachers in both classes were female, between 30 and 35 years of age, with a White Caucasian background. The teacher in the “dog-class” was the owner of the schooldog.

### Instruments

The following questionnaires regarding socio-emotional wellbeing and school experiences were answered by the children. For those with known low reading skills, questions were read aloud by the experimenter in small groups. Since this study focused on socio-emotional aspects and access to achievement data of pupils is more restricted, no data on grades or achievement test scores were collected.

#### The depression scale for children (depressionstest für kinder — DTK, Rossmann, 2005)

This standardized questionnaire consists of 55 items and assesses the current depressive state via the dimensions dysphoria/low self-esteem, agitated behavior, and tiredness/ other psychosomatic aspects of depression. It is validated for children in third to sixth grade.

#### The questionnaire on emotional and social experiences in school, grade 3-4 (fragebogen zur erfassung emotionaler und sozialer schulerfahrungen von grundschulkindern dritter und vierter klassen, FEESS 3-4, Rauer and Schuck, 2003)

The 90 items of the FEESS ask about children's attitudes toward and social experiences in school. This validated, standardized questionnaire consists of the following seven subscales: school-related self-concept, social integration, class climate, a positive attitude toward school, positive emotions related to learning, willingness to make an effort, and feeling accepted by the teacher.

#### The questionnaire on emotion regulation in children and juveniles (fragebogen zur erhebung der emotionsregulation bei kindern und jugendlichen, FEEL-KJ, Grob and Smolenski, 2009)

The FEEL-KJ is a 57-item-questionnaire for children and juveniles, which assesses the strategies used for the regulation of the emotions anxiety, sadness and anger. It asks about the *adaptive strategies:* goal oriented actions, distraction, improvement of mood, acceptance, forgetting, reattribution, and cognitive problem-solving. It also captures the *maladaptive strategies:* giving-up, aggressive behavior, withdrawal, devaluation of self, and perseveration. In addition, the *further strategies* emotional expression, seeking social support and emotion control are assessed.

Since it is difficult to capture such a complex phenomenon as emotion regulation, especially via a questionnaire, we employed this instrument assessing strategies of emotion regulation as the best approximation for obtaining information on emotion regulation itself.

#### Further instruments

In order to capture possible differences between the teachers which might influence the children's socioemotional experiences in school, which cannot be disentangled from the influence of the presence of the dog, it was attempted to collect data to assess personality differences between the two teachers of the control and intervention class. However, the personality questionnaire (NEO-FFI, German version, Borkenau and Ostendorf, 2008) was only answered by the teacher of the intervention class. Therefore, these data will not be reported here.

## Results

Scores of the two classes were compared at t1 via *T*-tests to screen for significant differences (*p* < 0.10) between the classes before the introduction of the schooldog. Repeated measures ANOVAs were used to assess differences in changes of the parameters between the classes. In case of significant differences between groups at t1, the score at t1 was entered as covariate. Results are reported as significant at a level of *p* < 0.05, and as tendencies at a level of *p* > 0.05 < 0.10. Scale scores were not calculated if a student missed more than 10% of the items of this scale; in case of less missing data the scale score was estimated from the average of the ≥90% of answered items.

### Depression

Overall depression as well as the subscales of the DTK (*dysphoria/low self-esteem, agitated behavior, and tiredness/ other psychosomatic aspects of depression*) did not differ between intervention class and control class at t1. Repeated measures ANOVAs did not reveal a significant change in depression scores for all children between t1 and t2, and also no effect of the presence of the schooldog-teacher-team (factor: class) on the changes in depression (subscales and the overall score of the DTK).

### Social and emotional experiences in school

Among the seven subscales of the FEESS, *t*-tests revealed only significantly higher scores in positive class climate in the dog-class (t1: *t* = 2.219, *df* = 43, *p* = 0.032). No significant change over time (t1 to t2) for the whole sample was observed on any of the FEESS-scales (repeated measures ANOVA; see Table 1).

**Table 1:** **Mean and standard deviation of FEESS for intervention-class (IC) and control class (CC) at t1 and t2 plus *p*-value of *t*-tests for differences between groups at t1**.

**FEESS**	**t1**					**t2**			
	**IC**		**CC**		**P**	**IC**		**CC**	
	***M***	***SD***	***M***	***SD***		***M***	***SD***	***M***	***SD***
Attitude	29.1	(12.9)	28.6	(9.6)	0.880	30.1	(13.4)	23.7	(11.4)
Pos. emotion	26.7	(9.2)	28.0	(5.8)	0.567	29.3	(8.8)	24.2	(7.6)
Accepted	33.2	(7.5)	31.6	(4.8)	0.483	33.9	(5.5)	32.5	(7.4)
Effort	32.8	(7.9)	33.4	(4.8)	0.740	32.5	(6.8)	29.6	(5.8)
Integration	26.0	(6.4)	25.3	(4.1)	0.671	26.8	(5.3)	24.9	(4.2)
Climate	26.6	(5.4)	23.0	(5.9)	0.033	26.3	(5.1)	22.8	(4.2)
Self-concept	35.3	(7.1)	36.9	(6.0)	0.385	34.2	(9.2)	35.4	(5.9)

**Figure 1:**
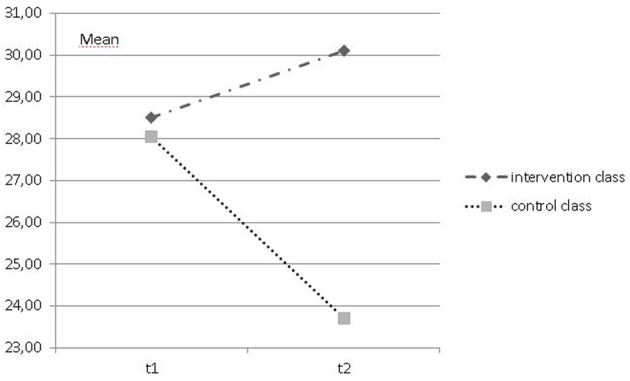
**Mean of FEESS-scale *positive attitude toward school* at t1 and t2 for intervention and control class**.

**Figure 2:**
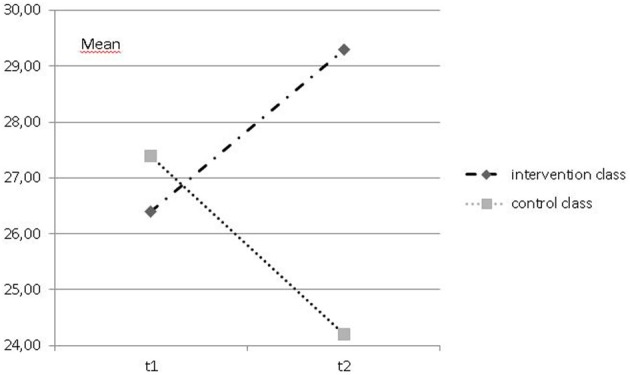
**Mean of FEESS-scale *positive emotions related to learning* at t1 and t2 for intervention and control class**.

Repeated measures ANOVAs revealed a significant positive change over time in the dog-class in comparison to the control class, whose scores decreased, for the scales *positive attitude toward school* (*F* = 10.769, *df* = 1, *p* = 0.002; *_p_*η^2^ = 0.216; see Figure 1), and *positive emotions related to learning* (*F* = 4.479, *df* = 1, *p* = 0.042, *_p_*η^2^ = 0.113; see Figure 2). Pre-post measure comparisons for each class showed a significant decline over time for positive attitude toward school for the control class (*f* = 5.599, *df* = 1, *p* = 0.029), and a change toward more positive attitudes toward school in the dog-class (*F* = 5.137, *df* = 1, *p* = 0.035). No significant changes within each class were found for the positive emotions related to learning. However, scores showed a similar trend, a decrease of positive emotions related to learning in the control class and an increase in the dog-class.

### Emotion regulation strategies

At t1, the dog-class students showed significantly higher scores in maladaptive strategies for emotion regulation (*t* = 1.734, *df* = 44, *p* = 0.090), and lower scores in adaptive strategies (*t* = −2.425, *df* = 44, *p* = 0.019). Repeated measures ANOVA revealed no significant differences between groups regarding the changes of adaptive, maladaptive and further emotion regulation strategies between t1 and t2. However, there was a significant effect of time for maladaptive strategies (*F* = 5.214, *df* = 1, *p* = 0.027). Even though group differences were not significant, the relatively large decrease in the maladaptive strategies in the dog-class, and the relatively large decrease in adaptive strategies in the control-class seem noteworthy (see Table 2).

**Table 2:** **Mean and standard deviation of the FEEL-KJ-scales adaptive and maladaptive emotion regulation for intervention-class (IC) and control class (CC) at t1 and t2**.

**FEEL-KJ**	**t1**				**t2**			
	**IC: *M***	***SD***	**CC: *M***	***SD***	**IC: *M***	***SD***	**CC: *M***	***SD***
Maladaptive	82.2	(16.8)	73.0	(19.2)	71.0	(18.8)	70.3	(13.4)
Adaptive	129.7	(29.0)	147.8	(19.5)	125.2	(42.1)	139.6	(27.0)

## Discussion

While the introduction of a schooldog into the classroom frequently aims at a reduction of negative attitudes or behaviors of few individuals (Beetz, 2012), our results show that it may also have some medium to strong effects on the entire class. In contrast to the control class, children in the dog-class significantly improved in their positive attitude toward school and their emotions related to learning. In contrast, the changes in the mean scores suggest a significant decline of positive attitudes toward school in the control-class, and a non-significant trend for a decline of positive emotions related to learning. This could be explained by the high pressure for academic achievement starting at the end of the third school year, since the future school career in this part of Germany depends primarily on the achievement during the fourth grade. The schooldog-teacher-team seemed not only able to buffer such a negative development in the dog-class, but even to enhance school-related attitudes and emotions.

No significant differences were found between the groups with regard to depression scores over the course of the school year. The 1-day per week presence of a schooldog in the classroom did obviously not significantly influence a normal range of depressive symptoms. The same was true for emotion regulation strategies. While mean scores suggested a positive change in maladaptive strategies in the dog-class and a negative change in adaptive strategies in the control class, this was not statistically significant.

Although we found statistically significant differences in the change of socioemotional school experiences and emotion regulation strategies between the two classes, it needs to be kept in mind that many other factors besides the dog, which are difficult to control, could have influenced these parameters. The main factors certainly are personality and interaction style of the teachers, but also of the different students in each class. The effects of the schooldog's presence cannot be disentangled from its combined influence together with the teacher via a research design as the one used in this study. However, experimental research comparing the same children with the same teacher with and without the dog present cannot capture long-term effects in socioemotional school experiences or competences. Only large scale studies with a sufficient number of classes with and without a schooldog present and additional data on the teaching style, personality and also human-dog-relationship of the teachers would allow a clearer interpretation of changes as effects of the schooldogs. But even then, the schooldog and teacher always affect the class climate as a team. Very likely the presence of the dog also influences the teacher in his or her behavior toward the students and stress regulation.

Furthermore, different dogs may have different effects, and also the frequency and duration of its presence may be related to the strength of positive effects. Also these factors could only be controlled for in a large-scale study with several dog-classes and control classes. Another approach to this research question would be experimental designs which allow controlling for some of these factors, e.g., employing the same teacher with and without the schooldog during the experiements. Also further behavior observations in presence and absence of the schooldog in the same classroom could produce more valuable insights on interactions between teacher and students and among students (see Kotrschal and Ortbauer, 2003). Overall, this uncontrolled influence of two different teachers and students' personality and interaction style in our study limits the validity of the findings. It is likely an influence of the individual teacher in combination with her schooldog in comparison to the teacher of the control class instead of the effect of the schooldog *per se*. Generalization to other schooldog-teacher-teams or interpretation of differences as effect of the dog itself is as limited as in most studies on this topic (see above).

Despite the study's limitations, the results presented above point to some positive correlates which might be attributed to the presence of the schooldog-teacher-team. The more positive socio-emotional experiences with regard to school and learning could be indirectly influenced by more positive interaction with peers and the teacher, but also with the dog itself. If stress reduction and promotion of positive social interactions indirectly affected these changes, or occurred at all, remains unknown. However, the social interactions in the classroom have a strong influence on all socio-emotional school experiences (Rauer and Schuck, 2003), and thus it seems plausible that this would be an underlying mechanism also here. Even though depressive mood was not significantly influenced it seems that the dog-teacher-team directly or indirectly promoted positive emotions, at least with regard to learning. This would be in accordance with the general positive effects of human-animal interaction on mood (Beetz et al., 2012b). A positive effect of the long-term presence of a schooldog on emotion regulation seems possible, but needs to be confirmed in further research, probably with a larger sample or with students with emotional and behavioral disorders, who usually show deficits in this area.

Since a prerequisite of effective social and cognitive learning is a positive mood and attitude, a schooldog, working as a team with his teacher, has the potential to support learning and educational goals by promoting these factors. Together with the findings of previous research on the reduction of depression and promotion of attention and empathy, the results of this study support the positive potential of schooldogs in the classroom. The documented effects parallel the experiences of teachers in Germany, Austria, and Switzerland who already work with a schooldog (Beetz, 2012). It can be expected that the trend of increasing numbers of schooldog-teacher-teams will continue, also because social and emotional disorders and negative school-related social experiences, even among students in regular schools, are increasing and different approaches that could counteract this development are needed.

### Conflict of interest statement

The author declares that the research was conducted in the absence of any commercial or financial relationships that could be construed as a potential conflict of interest.
